# Compartment-Specific CD138 Expression Defines an Aggressive Breast Cancer Phenotype with Distinct Transcriptomic Features

**DOI:** 10.3390/cancers18101539

**Published:** 2026-05-09

**Authors:** Kyoko Asai, Takahiro Hasebe, Masahiro Ohara, Masataka Hirasaki, Kazuo Matsuura, Hiroshi Ishiguro, Akihiko Osaki, Toshiaki Saeki

**Affiliations:** 1 Department of Breast Oncology, Saitama Medical University International Medical Center, Hidaka City 350-1298, Japan; oharamas0930@saitama-med.ac.jp (M.O.); m79kazuo@saitama-med.ac.jp (K.M.); ishiguro@saitama-med.ac.jp (H.I.); aosaki@saitama-med.ac.jp (A.O.); tsaeki@saitama-med.ac.jp (T.S.); 2 Department of Clinical Cancer Genomics, Saitama Medical University International Medical Center, Hidaka City 350-1298, Japan; hirasaki@saitama-med.ac.jp

**Keywords:** CD138, syndecan-1, breast cancer, tumor microenvironment, snoRNA, translational reprogramming, prognostic biomarker, RNA sequencing, immunohistochemistry, metastasis

## Abstract

**Background/Objectives**: CD138 (syndecan-1) is a cell-surface heparan sulfate proteoglycan involved in cell–matrix interactions and growth factor signaling, and it has been implicated in tumor progression. However, the clinical significance of compartment-specific CD138 expression in breast cancer remains unclear. In this study, we investigated the prognostic and transcriptomic features of compartment-specific CD138 expression in invasive breast cancer. **Methods**: We performed an integrated analysis of immunohistochemistry and RNA sequencing of 111 invasive ductal carcinoma specimens. Tumors were classified into four groups according to CD138 expression in the tumor and stromal compartments. Clinicopathological data and survival outcomes were obtained from medical records, and transcriptomic profiles were analyzed using RNA sequencing. **Results**: The tumor-positive/stroma-negative phenotype (Group 1) was associated with poorer recurrence-free survival than the other phenotypes. According to multivariable Cox regression analysis, Group 1 remained an independent prognostic factor after adjustment for age, lymph node status, and Ki-67 index (hazard ratio, 5.493; *p* = 0.0028). Group 1 was also associated with lymph node metastasis and HER2 expression. All brain metastases occurred in Group 1, although the number of events was low. Transcriptomic profiling identified the upregulation of small nucleolar RNAs in Group 1 tumors, with the enrichment of pathways related to ribosome biogenesis, RNA processing, and translational regulation. **Conclusions**: Compartment-specific CD138 expression identifies an aggressive breast cancer phenotype with distinct transcriptomic features. This phenotype may have prognostic value and warrants validation using larger, independent cohorts.

## Simple Summary

CD138 (syndecan-1) is a cell-surface molecule involved in cell adhesion and growth factor signaling. Its clinical significance in breast cancer remains unclear, particularly when its expression is assessed separately in the tumor and stromal compartments. We examined compartment-specific CD138 expression in tumor and stromal cells in 111 invasive breast cancers. Tumors with CD138-positive tumor cells and CD138-negative stromal cells were associated with poorer recurrence-free survival and adverse clinicopathological features, including lymph node metastasis and HER2 positivity. All brain metastases occurred in this phenotype, although the number of events was low. RNA-seq analysis showed the upregulation of small nucleolar RNAs and enrichment of pathways related to ribosome biogenesis, RNA processing, and translational regulation. These findings suggest that compartment-specific CD138 expression may help to identify an aggressive breast cancer phenotype with potential prognostic relevance.

## 1. Introduction

Breast cancer is a biologically heterogeneous disease characterized by diverse tumor cell-intrinsic programs and dynamic interactions with the tumor microenvironment (TME) [1,2]. Molecular classification based on hormone receptor (HR) and human epidermal growth factor receptor 2 (HER2) status has improved prognostic stratification and therapeutic decision-making in breast cancer. Nevertheless, a considerable proportion of patients still develop recurrence, underscoring the need for additional biomarkers that reflect tumor–stromal crosstalk and post-transcriptional regulatory mechanisms involved in tumor progression.

Several cluster of differentiation (CD) molecules have been implicated in breast cancer progression and tumor heterogeneity. Among them, CD44 and CD24 are well established markers associated with cancer stemness, tumor aggressiveness, and metastatic potential. CD44, a receptor for hyaluronic acid, mediates tumor cell adhesion and migration and has been used for targeted drug delivery in CD44-positive cancer cells [3]. In addition, the CD44/CD24 expression profile has been extensively studied in breast cancer, where CD44-positive/CD24-negative phenotypes are associated with aggressive clinical behavior and poor prognosis [4]. These findings highlight the importance of CD molecule expression patterns in defining biologically distinct tumor subtypes and underscore the relevance of investigating other CD markers, including CD138, in breast cancer progression.

Syndecan-1 (CD138) is a transmembrane heparan sulfate proteoglycan involved in cell–cell and cell–matrix interactions, growth factor signaling, and extracellular matrix (ECM) organization [5,6]. In breast cancer, CD138 expression has been observed in both tumor and stromal cells, particularly cancer-associated fibroblasts [7]. However, studies evaluating the prognostic significance of CD138 expression in breast cancer have yielded inconsistent results; some studies have associated CD138 expression with poor clinical outcomes, whereas others reported more favorable outcomes. These discrepancies may partly reflect differences in evaluation methods, including whether CD138 expression in the tumor and stromal compartments was assessed separately or collectively [8,9].

Recent immunohistochemical studies suggest that compartment-specific CD138 expression may have distinct biological implications. It has been suggested that stromal CD138 expression exerts tumor-modulating effects, whereas CD138 expression restricted to tumor epithelial cells may be associated with a more aggressive phenotype [7,8]. Despite these observations, the molecular basis of compartment-specific CD138 expression patterns remains unclear. In particular, the intracellular regulatory programs associated with CD138 expression, especially those related to gene expression control and protein synthesis, have not been fully elucidated.

RNA sequencing (RNA-seq) enables comprehensive profiling of both coding and non-coding RNAs and has revealed that small nucleolar RNAs (snoRNAs), traditionally regarded as housekeeping molecules involved in ribosomal RNA (rRNA) modification, play broader roles in cancer biology. snoRNAs are broadly classified into the H/ACA box and C/D box families, which guide the pseudouridylation and 2′-O-methylation of rRNA, thereby influencing the ribosome structure and translational fidelity [10,11,12,13,14].

Recent studies have demonstrated that snoRNAs contribute to tumor progression, metastasis, and therapeutic resistance across multiple cancer types, including breast cancer [15,16,17]. Furthermore, alterations in snoRNA expression are often coordinated through their host genes and may reflect broader changes in RNA processing and translational regulatory networks [18].

These findings suggest that post-transcriptional reprogramming mediated by snoRNAs may promote tumor aggressiveness by enabling the selective translation of oncogenic transcripts. However, whether CD138-associated tumor phenotypes are linked to such translational control programs remains largely unexplored.

In this study, we aimed to clarify the clinical significance of compartment-specific CD138 expression in invasive breast cancer and evaluate its potential as a prognostic biomarker. We classified tumors according to the immunohistochemical patterns of CD138 expression in the tumor epithelial and stromal compartments and assessed their associations with clinicopathological features and patient outcomes. To further investigate the biology underlying CD138-associated tumor states, we integrated this immunohistochemical classification with transcriptomic profiling using RNA-seq.

Through this integrative analysis, we identified snoRNAs and host genes associated with CD138-defined tumor states and explored the underlying translational regulatory pathways. This approach provides novel insights into the molecular programs associated with compartment-specific CD138 expression and highlights a potential link between cell-surface signaling and post-transcriptional regulation in breast cancer. Therefore, elucidating the connection between CD138 expression patterns and snoRNA-mediated regulatory networks may provide new insights into the molecular basis of tumor aggressiveness.

## 2. Materials and Methods

### 2.1. Patients and Tumor Specimens

A total of 111 invasive ductal carcinoma (IDC) specimens were obtained from patients who underwent surgical resection at our institution between January 2007 and October 2015. Tumors were classified according to the Union for International Cancer Control’s TNM classification system. Resected specimens were fixed in 10% neutral-buffered formalin and processed for routine histopathological examinations.

Estrogen receptor (ER), progesterone receptor (PgR), and human epidermal growth factor receptor 2 (HER2) statuses were determined in accordance with the American Society of Clinical Oncology/College of American Pathologists (ASCO/CAP) guidelines [19,20]. The Ki-67 labeling index was defined as the percentage of invasive tumor cells showing positive nuclear staining among the total number of tumor cells counted using MIB-1 (mouse monoclonal antibody; Dako).

Clinicopathological data, including age, tumor size (T stage), lymph node status (N stage), histological grade (HG), Ki-67 index, HR status, HER2 status, recurrence or metastasis, and adjuvant therapy, were collected from medical records (Table 1).

### 2.2. Immunohistochemistry

**Figure 1:**
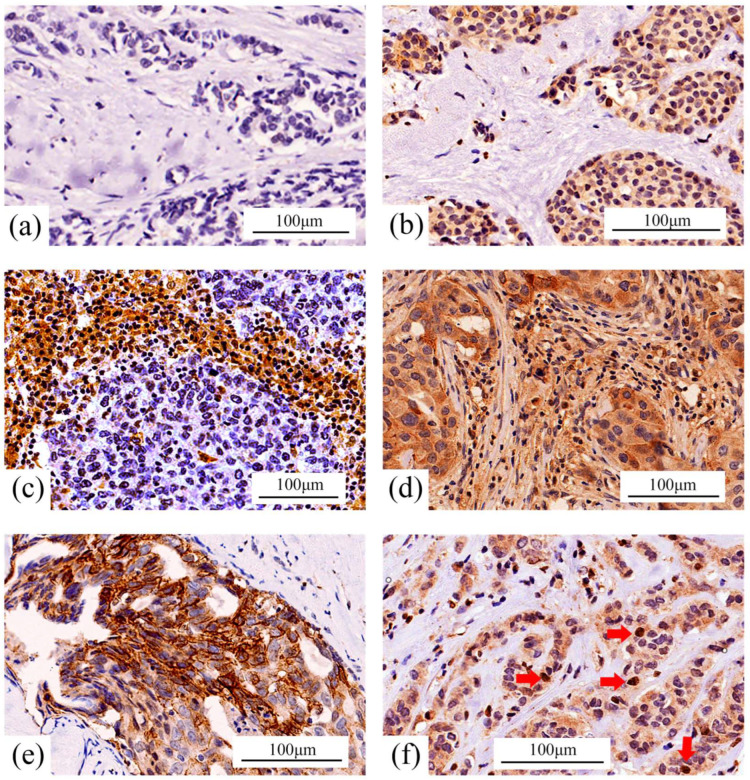
The representative immunohistochemical staining patterns of CD138. Representative CD138 immunohistochemical staining patterns in the tumor epithelial and stromal compartments are shown: (**a**) Group 0: tumor-negative/stroma-negative; (**b**) Group 1: tumor-positive/stroma-negative; (**c**) Group 2: tumor-negative/stroma-positive; (**d**) Group 3: tumor-positive/stroma-positive; (**e**) representative membranous CD138 staining in tumor cells; (**f**) representative nuclear CD138 staining in tumor cells (red arrows).

Immunohistochemical (IHC) staining was performed using a rabbit polyclonal anti-CD138 antibody (Proteintech; dilution 1:750), a Dako EnVision+ System-HRP-labeled polymer anti-rabbit secondary antibody, and a Dako Liquid DAB+ Substrate Chromogen System (Figure 1).

IHC staining for CD138 was independently evaluated by two experienced observers blinded to the clinicopathological data. Discrepant cases were jointly reviewed to reach a consensus.

CD138 expression was assessed separately in tumor epithelial cells and the stromal compartments. A semi-quantitative scoring system based on staining intensity and the proportion of positive cells/area was applied according to previously reported methods [21,22] and with reference to a tissue microarray-based scoring approach described by Lennartz et al. [23], with minor modifications.

The staining intensity was categorized as 0 (negative), 1+ (weak), 2+ (moderate), or 3+ (strong), and the proportion of positive tumor cells or the stromal area was recorded as a percentage (0–100%).

To define binary classification, the staining intensity scores were further evaluated using receiver operating characteristic (ROC) curve analysis to determine the optimal cutoff values. Based on this analysis, tumor cell expression was classified as negative (scores 0–1) or positive (scores 2–3), whereas stromal expression was classified as negative (score 0) or positive (scores 1–3). Accordingly, the tumors were categorized into four groups: Group 0 (tumor [−], stroma [−]), Group 1 (tumor [+], stroma [−]), Group 2 (tumor [−], stroma [+]), and Group 3 (tumor [+], stroma [+]).

The detailed criteria for the classification of CD138 expression are provided in Supplementary Table S1.

Based on the staining patterns (Table 2) observed in the tumor epithelial and stromal compartments, the cases were classified into four groups (Table 3).

### 2.3. RNA-Seq

A total of 111 breast cancer cases were included in the RNA-seq analysis. Of these, 17 cases from project PRJDB37924 were previously analyzed in an earlier study [25]. An additional 78 cases from the same project (PRJDB37924) were analyzed using the same protocol, and 16 newly sequenced cases (PRJDB40174) were included, yielding 111 samples for analysis.

For the 16 newly sequenced cases, total RNA was extracted from formalin-fixed paraffin-embedded (FFPE) tissue using the RNeasy FFPE Kit (QIAGEN, Hilden, Germany). The overall analytical pipeline followed the methodology described previously [25]. Gene expression levels were quantified as transcripts per million (TPM) for downstream analysis.

The gene expression values were log_2_-transformed as log_2_(TPM + 1). Genes with an absolute difference in the mean log_2_(TPM + 1) expression of ≥1 between groups and a *p*-value < 0.05 were defined as differentially expressed genes (DEGs). Differential expression analysis was performed across the CD138 expression groups defined in Table 3 to identify the CD138-associated RNAs.

Statistical thresholds were defined according to the purpose of each analysis. For the exploratory identification of DEGs, a threshold of *p* < 0.05 was applied to maintain sensitivity. For downstream analyses, including enrichment analysis, false discovery rate (FDR)-adjusted criteria were used to control for multiple testing.

A volcano plot was generated to visualize the distribution of differentially expressed RNAs, with log_2_ fold change and statistical significance.

The RNA-seq datasets analyzed in this study are publicly available in the DNA Data Bank of Japan Sequence Read Archive under the accession numbers PRJDB37924 and PRJDB40174.

### 2.4. Survival Analysis

Recurrence-free survival (RFS) was estimated using the Kaplan–Meier method. Patients were followed up from the date of surgery until the first documented recurrence or the date of their last follow-up. Recurrence or metastasis was defined as an event, whereas patients without recurrence were censored at their last follow-up.

To evaluate the relationship between transcript-level and protein-level CD138 expression, tumors were categorized according to CD138 transcript abundance derived from RNA-seq using TPM values. The cutoff value was determined using ROC analysis, with recurrence status as the classification endpoint. The association between the TPM-based CD138 expression groups and immunohistochemically defined CD138 expression groups was assessed using the chi-square test.

### 2.5. snoDB 2.0 Annotation and Host Gene Extraction

snoRNAs were annotated using the snoDB 2.0 database [26], and their corresponding host genes were identified. Host gene attributes, including box class (H/ACA or C/D), canonical rRNA modification sites, predicted modification type (pseudouridylation [Ψ] or 2′-O-methylation), and family or single-copy status, were retrieved from snoDB 2.0. Duplicate host genes were removed before enrichment analysis to avoid redundancy in the gene set.

### 2.6. Functional Enrichment Analysis

Functional enrichment analysis of DEGs was performed using the Database for Annotation, Visualization, and Integrated Discovery (DAVID) to identify enriched Gene Ontology (GO) Biological Process terms and Kyoto Encyclopedia of Genes and Genomes (KEGG) pathways.

Functional enrichment analysis of snoRNA host genes derived from CD138-associated transcriptomic signatures was performed using the clusterProfiler package in R [27]. GO Biological Process terms and KEGG pathways were evaluated using a complete set of human genes as a background reference. Multiple testing correction was performed using the Benjamini–Hochberg method, and an FDR of <0.05 was considered statistically significant.

### 2.7. CD138 ssGSEA Signature Scoring

A curated gene set representing the CD138-positive expression program was constructed from differentially expressed protein-coding genes identified between CD138-positive and CD138-negative tumors based on immunohistochemical classification. The CD138-positive gene signature comprised S100A7, CD24, and GLYATL2, which were upregulated in tumors with a CD138-positive phenotype. The CD138-negative signature included RERG, SLC39A6, NAT1, SCUBE2, PIP, NPY1R, SLC7A2, GRIA2, FSIP1, PTPRT, and SERPINA3, which were enriched in CD138-negative tumors.

Single-sample gene set enrichment analysis (ssGSEA) was performed using the GSVA package in R [28] on log_2_(TPM + 1)-transformed expression values derived from the RNA-seq dataset. ssGSEA computes an enrichment score for each sample based on the ranked expression levels of genes within a predefined gene set.

CD138-positive ssGSEA scores were used to estimate the activity of the CD138-associated transcriptional program in each tumor sample. Scores were compared among groups defined based on immunohistochemical CD138 expression patterns, and correlations with S100A7 and CD24 expression were evaluated.

### 2.8. Derivation of the snoRNA Module Score

snoRNAs significantly enriched in CD138-positive tumors (Group 1, tumor-positive/stroma-negative) were extracted to generate a snoRNA signature set. For each sample, the snoRNA module score was calculated as the mean TPM of all the snoRNAs included in the signature set.

### 2.9. Statistical Analysis

Differences in ssGSEA scores between groups were evaluated using the Mann–Whitney U test, and correlations between ssGSEA scores and transcript expression levels were assessed using Spearman rank correlation coefficients.

Associations between the CD138 expression groups and clinicopathological variables were evaluated using the chi-square test or the Fisher exact test, as appropriate. The Fisher exact test was applied when the expected cell count was <5. The analyzed variables included age (<40 years), tumor size (T stage), lymph node status (N stage), HG, Ki-67 index, HR status, HER2 status, brain metastasis, and adjuvant therapies (radiotherapy, chemotherapy, endocrine therapy, and anti-HER2 therapy).

RFS was analyzed using the Kaplan–Meier method, and differences between the groups were assessed using the log-rank test. Prognostic factors were evaluated using Cox proportional hazards regression. The multivariable models included age, tumor size (T stage), lymph node status (N stage), HG, Ki-67 index, HR status, HER2 status, CD138 expression group (Group 1), and treatment variables selected on the basis of clinical relevance and univariate results.

Continuous variables, including the ssGSEA and snoRNA module scores, were standardized to z-scores before multivariable modeling. The proportional hazards assumption was assessed using the Schoenfeld residuals. Model performance was evaluated using the concordance index (C-index).

All statistical tests were two-sided, and *p*-values < 0.05 were considered statistically significant. All analyses were performed using R software (v4.3.2).

## 3. Results

### 3.1. CD138 Immunohistochemical Expression Patterns and Clinical Classification

Immunohistochemical staining for CD138 revealed heterogeneous expression across the tumor epithelial and stromal compartments (Figure 1). The evaluation, conducted according to the criteria reported by Kind et al. [21] and Choi et al. [22] (Supplementary Table S1), is summarized in Table 2. When evaluated individually, membranous tumor cell expression, stromal expression, and nuclear tumor cell expression of CD138 were not significantly associated with RFS.

Although previous studies by Kind et al. [21] and Choi et al. [22] focused primarily on membranous and stromal expression, we also observed the nuclear localization of CD138 in a subset of tumor cells. Nuclear CD138 expression in breast cancer has rarely been described previously.

Based on the evaluation frameworks of Kind et al. [21] and Choi et al. [22], we classified the cases using an integrated tumor/stroma CD138 expression system (Table 3). Tumors were categorized into four groups: tumor-negative/stroma-negative (Group 0), tumor-positive/stroma-negative (Group 1), tumor-negative/stroma-positive (Group 2), and tumor-positive/stroma-positive (Group 3).

**Figure 2:**
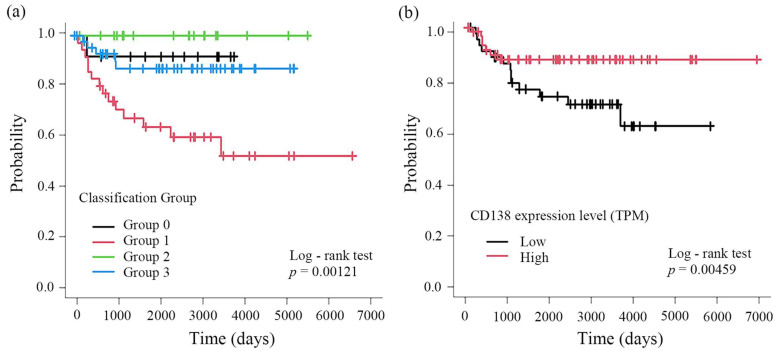
Recurrence-free survival according to CD138 expression patterns. (**a**) Kaplan–Meier curves of recurrence-free survival (RFS) according to CD138 immunohistochemical classification: Group 0, tumor-negative/stroma-negative; Group 1, tumor-positive/stroma-negative; Group 2, tumor-negative/stroma-positive; Group 3, tumor-positive/stroma-positive. Group 1 showed significantly poorer RFS than the other groups (*p* = 0.00121). (**b**) Kaplan–Meier curves of RFS stratified by CD138 transcript expression levels (TPM). Patients were dichotomized using a cutoff value of 50.56, determined via receiver operating characteristic (ROC) analysis (*p* = 0.0459). Abbreviations: RFS, recurrence-free survival; TPM, transcripts per million; ROC, receiver operating characteristic.

Kaplan–Meier survival analysis showed that Group 1 (tumor-positive/stroma-negative) had significantly poorer RFS than the other groups (Figure 2a). These findings indicate that RFS differs according to the compartment-specific CD138 expression pattern.

In contrast, Kaplan–Meier analysis based on CD138 transcript expression levels derived from RNA-seq [log_2_(TPM + 1)] showed that tumors with lower overall CD138 expression had significantly worse RFS (Figure 2b).

To evaluate the relationship between the CD138 IHC classification and transcript-level expression, a chi-square test was performed, and no significant association was observed (χ^2^ = 0.254, df = 3, *p* = 0.968) (Table 4).

This discrepancy between the immunohistochemical and transcript-level findings may reflect differences in the measurement approach. RNA-seq quantifies CD138 expression across the entire tumor tissue, including stromal components, whereas IHC permits compartment-specific discrimination between tumor epithelial and stromal cells. These results show that the prognostic association of CD138 expression differed according to the compartment in which it was expressed.

### 3.2. Identification of CD138-Associated Differentially Expressed RNAs

**Figure 3:**
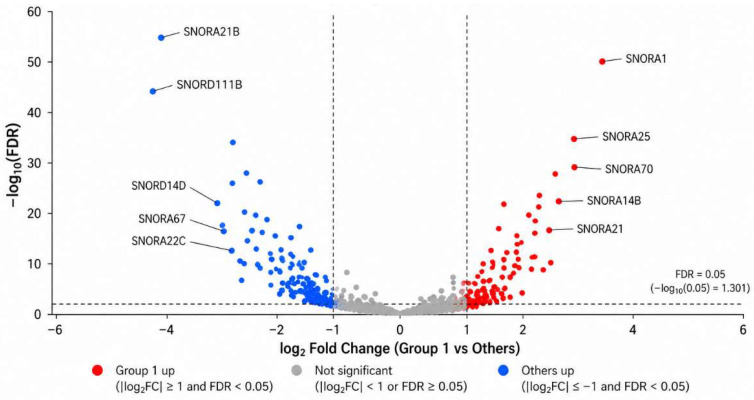
Volcano plot of differentially expressed snoRNAs (Group 1 vs. Others). The x-axis represents log_2_ fold change (Group 1 vs. Others), and the y-axis represents −log_10_(FDR). Dashed vertical lines indicate |log_2_FC| = 1, and the horizontal dashed line indicates FDR = 0.05.

To provide an overview of the differential expression profile associated with CD138-defined tumor states, a volcano plot was generated (Figure 3). The plot illustrates the distribution of RNAs according to log_2_ fold change and statistical significance. Group 1 tumors exhibited a distinct expression pattern characterized by the upregulation of multiple snoRNAs and downregulation of various non-coding and protein-coding RNAs.

Differential expression analysis of RNA-seq data comparing Group 1 with Groups 0, 2, and 3 identified a set of RNAs associated with the tumor-positive/stroma-negative phenotype (Table 5).

Group 1 tumors showed significant upregulation of multiple snoRNAs, including SNORA21, SNORA1, SNORA25, SNORD14D, SNORA70, SNORA14B, SNORA22C, SNORD111, SNORD111B, SNORA21B, and SNORA67 (Table 5a). Among the protein-coding genes, S100A7, GLYATL2, and CD24 were significantly upregulated in Group 1 tumors (Table 5a). Several ncRNAs were significantly downregulated in Group 1 tumors (Table 5b). These included U3 snoRNA, multiple Y RNAs, and several signal recognition particle-related RNAs (Table 5b). Multiple antisense long non-coding RNAs (lncRNAs), including GATA3-AS1, PDCD4-AS1, and SIAH2-AS1, were also significantly decreased in Group 1 tumors (Table 5b). Among the protein-coding genes, SCUBE2, NAT1, RERG, and PIP showed significantly reduced expression in Group 1 tumors (Table 5b). Overall, the tumor-positive/stroma-negative phenotype was associated with the upregulation of multiple snoRNAs and downregulation of several non-coding and protein-coding RNAs.

### 3.3. Identification of CD138-Associated snoRNAs

S100A7 and CD24 were among the transcripts upregulated in Group 1 tumors [29,30,31,32]. These results indicate that the tumor-positive/stroma-negative CD138 expression pattern was associated with the differential expression of both protein-coding genes and snoRNAs.

### 3.4. Functional Annotation of snoRNA Host Genes

Differentially expressed snoRNAs were annotated using the snoDB 2.0 database. The log_2_ fold change (logFC) and FDR from the differential expression analysis conducted for each snoRNA are shown in Table 6. Annotation using snoDB 2.0 identified the corresponding protein-coding host genes for the differentially expressed snoRNAs. These snoRNAs belong to both the H/ACA box and C/D box subclasses and are associated with diverse rRNA modification targets, including pseudouridylation and 2′-O-methylation sites.

**Figure 4:**
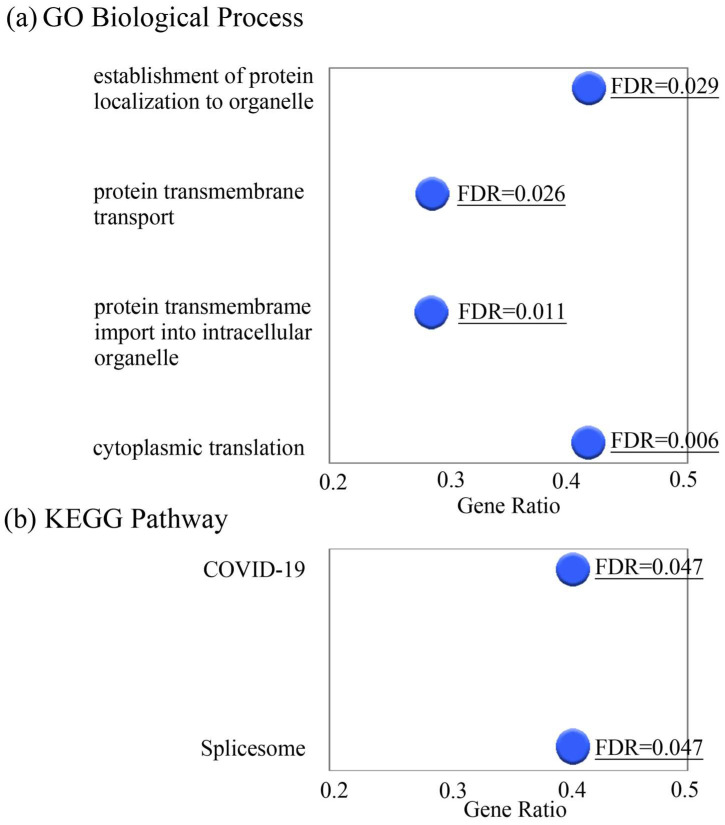
Functional enrichment analysis of snoRNA host genes. Gene Ontology (GO) Biological Process (BP) and Kyoto Encyclopedia of Genes and Genomes (KEGG) pathway enrichment analyses were performed for the host genes of differentially expressed snoRNAs. The significantly enriched pathways (FDR < 0.05) were mainly related to RNA processing and protein translation. (**a**) GO BP enrichment of snoRNA host genes. (**b**) KEGG pathway enrichment analysis of snoRNA host genes. Abbreviations: GO, Gene Ontology; BP, Biological Process; KEGG, Kyoto Encyclopedia of Genes and Genomes; FDR, false discovery rate.

Several snoRNAs belong to paralogous gene families with multiple genomic copies. GO and KEGG pathway enrichment analyses of snoRNA host genes showed significant enrichment of pathways related to ribonucleoprotein complex biogenesis, RNA processing, translational regulation, and spliceosome function (Figure 4).

Overall, Group 1 tumors showed enrichment in snoRNA-associated pathways related to ribosome biogenesis, RNA processing, and translational control. These findings indicate that compartment-specific CD138 expression is associated with a distinct transcriptomic profile.

### 3.5. Quantification of the CD138-Associated Transcriptional Program

**Figure 5:**
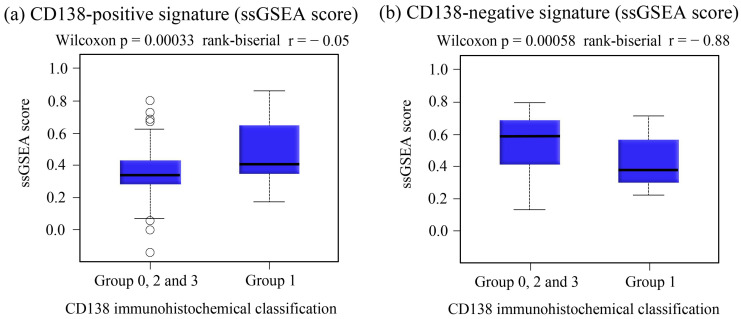
The association between CD138 immunohistochemical status and transcriptional signatures. Comparison of ssGSEA scores derived from CD138-positive and CD138-negative transcriptional signatures according to CD138 immunohistochemical status. The ssGSEA score represents the enrichment level of the predefined CD138-associated gene set in each tumor sample. The CD138-positive gene signature consisted of S100A7, CD24, and GLYATL2, whereas the CD138-negative signature included RERG, SLC39A6, NAT1, SCUBE2, PIP, NPY1R, SLC7A2, GRIA2, FSIP1, PTPRT, and SERPINA3 genes. (**a**) Tumors with a CD138-positive immunohistochemical status had significantly higher ssGSEA scores for the CD138-positive signature than CD138-negative tumors. (**b**) Tumors lacking CD138 expression had significantly higher scores for the CD138-negative signature. Statistical comparisons were performed using the Wilcoxon rank-sum test. Each point represents an individual tumor sample. Boxes indicate the interquartile range (IQR), center lines indicate the median, and whiskers represent values within 1.5 × IQR.

To evaluate the transcriptional state associated with CD138 expression, we calculated an ssGSEA-based CD138 signature from the tumor RNA-seq profiles. The snoRNA module score derived from CD138-associated snoRNAs also differed among the groups and was significantly higher in Group 1 tumors (tumor-positive/stroma-negative) (Figure 5). These results indicate that Group 1 tumors had higher CD138-related transcriptional and snoRNA module scores.

### 3.6. Association Between the CD138 Signature and Gene Expression Markers

**Figure 6:**
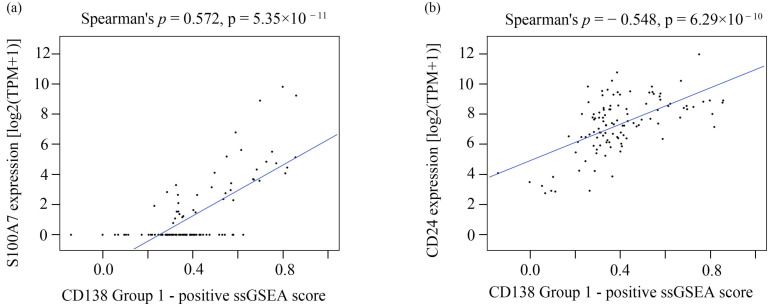
Correlation between CD138 ssGSEA scores and S100A7/CD24 expression. Scatter plots showing the association between the CD138-positive (Group 1) transcriptional signature score and the expression levels of (**a**) S100A7 and (**b**) CD24 [log_2_(TPM + 1)]. Spearman correlation coefficients and *p*-values are shown. Abbreviations: ssGSEA, single-sample gene set enrichment analysis; TPM, transcripts per million.

Spearman correlation analysis showed positive correlations between the Group 1 signature score and S100A7 expression (Spearman ρ = 0.572, *p* = 5.35 × 10^−11^) and between the Group 1 signature score and CD24 expression (Spearman ρ = 0.548, *p* = 6.29 × 10^−10^) (Figure 6). These findings indicate that the CD138-positive signature score was positively correlated with S100A7 and CD24 expression.

### 3.7. Relationship Between CD138 Transcriptional Activity and the snoRNA Program

**Figure 7:**
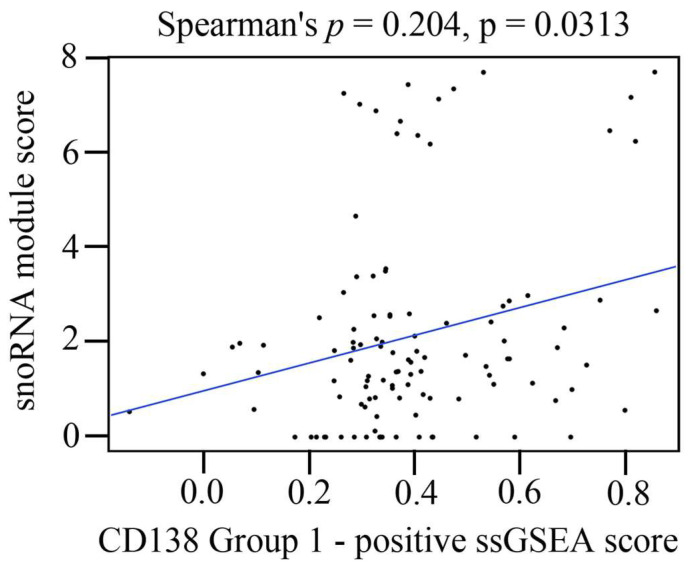
The relationship between CD138 ssGSEA scores and snoRNA module scores. A scatter plot comparing the CD138-positive (Group 1) ssGSEA score and the snoRNA module score for each patient. A positive correlation was observed between the CD138-positive ssGSEA and snoRNA module scores. Abbreviations: ssGSEA, single-sample gene set enrichment analysis.

The snoRNA module score was positively correlated with the CD138-positive ssGSEA score (Figure 7). This association indicates that higher CD138 signature scores co-occurred with higher snoRNA module scores.

### 3.8. Association Between Group 1 CD138 Expression Group and Clinicopathological Factors

Next, we examined the association between Group 1 CD138 expression status and clinicopathological variables (Table 7).

Group 1 was significantly associated with several adverse clinicopathological features. HER2 positivity was more frequently observed in Group 1 than in the other groups (40.0% vs. 13.2%, *p* = 0.002). Anti-HER2 therapy was administered more frequently in Group 1 than in the other groups (28.6% vs. 7.9%, *p* = 0.001). Lymph node metastasis was also more common in Group 1 than in the other groups (48.6% vs. 19.7%, *p* < 0.001).

Among the 111 patients, four developed brain metastases, and all four cases belonged to Group 1. Brain metastases were observed only in Group 1 and were significantly associated with CD138 expression status (11.4% vs. 0%, *p* = 0.0098; Fisher exact test).

A high HG was more frequent in Group 1 than in the other groups (48.6% vs. 31.6%, *p* = 0.036), whereas HR positivity was lower (51.4% vs. 71.1%, *p* = 0.044).

The results for tumor size (T stage), Ki-67 index, transcript-level CD138 expression (TPM), chemotherapy, and radiotherapy did not differ significantly between Group 1 and the other groups.

Overall, Group 1 was associated with HER2 positivity, lymph node metastasis, high HG, low HR positivity, and brain metastases.

### 3.9. Prognostic Impact of Clinicopathological Factors and CD138 Expression

To evaluate the prognostic factors associated with RFS, univariable and multivariable Cox proportional hazards analyses were performed (Table 8).

In the univariable analysis, younger age (HR = 0.273, 95% CI: 0.099–0.752, *p* = 0.012), advanced tumor size (T stage) (HR = 3.882, 95% CI: 1.518–9.927, *p* = 0.0046), lymph node metastasis (N stage) (HR = 4.581, 95% CI: 1.954–10.74, *p* < 0.001), a high Ki-67 index (HR = 3.025, 95% CI: 1.020–8.969, *p* = 0.046), and the CD138 tumor-positive/stroma-negative phenotype (Group 1) (HR = 4.877, 95% CI: 1.994–11.93, *p* < 0.001) were associated with worse RFS. Endocrine therapy was associated with better RFS in the univariable analysis (HR = 0.393, 95% CI: 0.165–0.937, *p* = 0.035).

In the multivariable analysis, lymph node metastasis (HR = 4.763, 95% CI: 1.717–13.21, *p* = 0.0027), a high Ki-67 index (HR = 3.789, 95% CI: 1.052–13.65, *p* = 0.0416), and the CD138 tumor-positive/stroma-negative phenotype (Group 1) (HR = 6.13, 95% CI: 2.081–18.06, *p* = 0.0010) remained independently associated with worse RFS.

Tumor size, HG, HR status, HER2 status, and treatment variables were not independently associated with RFS in the multivariable model.

The CD138 tumor-positive/stroma-negative phenotype remained independently associated with recurrence after adjusting for lymph node status and Ki-67 index. These findings indicate that compartment-specific CD138 expression is associated with RFS after adjustment for conventional clinicopathological factors.

Overall, the tumor-positive/stroma-negative CD138 phenotype was independently associated with worse RFS in this cohort.

## 4. Discussion

Breast cancer is a biologically heterogeneous disease shaped by both tumor cell-intrinsic programs and dynamic interactions with the tumor microenvironment [2,33]. In this study, compartment-specific CD138 expression identified a clinically distinct subgroup of invasive breast cancer associated with poor RFS and transcriptomic features related to snoRNA expression, RNA processing, and translational regulation. The tumor-positive/stroma-negative CD138 phenotype (Group 1) remained independently associated with poorer RFS after adjusting for lymph node status and Ki-67 index.

An important observation of this study is that CD138 expression in the tumor and stromal compartments should be interpreted together rather than in isolation. Previous studies have often evaluated CD138 positivity in either tumor cells or stromal cells alone, which may have contributed to the identification of inconsistent prognostic findings. By integrating tumor and stromal CD138 expression into a single classification framework, we identified a subgroup characterized by tumor-positive/stroma-negative expression, which was associated with worse clinical outcomes. These findings raise the possibility that stromal and tumor cell CD138 expression have different biological and prognostic associations. Kind et al. reported associations between combined tumor–stromal CD138 expression and unfavorable clinicopathological features [21]. Our findings further suggest that the spatial distribution of CD138 expression may be more informative than overall positivity.

Transcript-level analysis showed that lower CD138 mRNA expression was associated with a poorer prognosis but did not correlate with the immunohistochemically defined Group 1 phenotype. This discrepancy suggests that transcript-level CD138 expression and spatial protein expression capture different aspects of tumor biology and supports the value of using compartment-specific protein assessment when investigating tumor microenvironment-related biomarkers.

CD138 is a transmembrane heparan sulfate proteoglycan involved in growth factor signaling, ECM organization, and cell–cell communication. In stromal fibroblasts, CD138 contributes to ECM integrity and cell adhesion through interactions with ECM components and may influence tumor cell motility and invasion [34,35]. CD138 has also been reported to regulate focal adhesion dynamics and cell migration through its interactions with ECM ligands and growth factors [35,36]. Reduced stromal CD138 expression may alter the structural and signaling integrity within the tumor microenvironment and potentially facilitate tumor invasion and recurrence [36]. Conversely, persistent CD138 expression in tumor cells may enhance growth factor signaling by retaining ligands such as VEGF fibroblast growth factor (FGF), hepatocyte growth factor (HGF), and vascular endothelial growth factor (VEGF) through heparan sulfate chains, promoting the activation of the MAPK and PI3K/Akt pathways [36].

At the transcriptomic level, Group 1 tumors showed enrichment of multiple snoRNAs belonging to both the H/ACA and C/D box subclasses, linked to diverse rRNA modification sites. These findings are consistent with the alterations in rRNA-related regulatory programs identified in Group 1 tumors. Although snoRNAs were historically viewed as housekeeping factors involved in ribosome biogenesis, accumulating evidence indicates that they also participate in cancer-related translational regulation [11,37,38]. Through rRNA modification, snoRNAs may influence ribosome structure and function, thereby enabling the selective translation of specific transcripts and contributing to ribosome heterogeneity. snoRNAs have also been implicated in cellular stress responses and tumor progression [37,39]. Our findings extend this concept by linking snoRNA-associated transcriptomic changes to a clinically aggressive breast cancer phenotype defined by compartment-specific CD138 expression [40,41]. These observations are more consistent with broader translational regulatory changes than with the effects of any single snoRNA. In addition, the association between CD138 expression and snoRNA-related transcriptomic alterations observed in this study should be interpreted primarily as correlative in nature. Further mechanistic studies are required to establish causality and elucidate the underlying biological pathways linking CD138 expression to translational reprogramming.

Functional enrichment analysis of snoRNA host genes supported this interpretation by showing the enrichment of pathways involved in RNA processing, ribonucleoprotein complex assembly, spliceosome function, and protein translation. Several host genes identified in this study, including RPL23, RPL10, EIF4A1, and TAF1D, are involved in ribosome assembly and translation initiation, processes often upregulated in cancer to support protein synthesis and stress adaptation [42,43,44,45,46]. Additional host genes, such as HSPA8, TOMM20, and CCT6P3, are involved in protein homeostasis, mitochondrial function, and molecular chaperone activity, which may support tumor cell survival and stress adaptation [47,48,49].

In addition to snoRNA upregulation, multiple ncRNAs were downregulated in Group 1 tumors, including U3 snoRNA, Y RNAs, and signal recognition particle-related RNAs. As U3 snoRNA plays an essential role in 18S rRNA processing and ribosome biogenesis, its reduced expression may reflect altered ribosome assembly in Group 1 tumors. Such alterations are consistent with the concept of specialized ribosomes, in which changes in ribosomal composition may influence the selective translation of specific mRNA transcripts. Decreased expression of several antisense lncRNAs, including GATA3-AS1, PDCD4-AS1, and SIAH2-AS1, may also reflect the disruption of differentiation-associated transcriptional programs.

Consistent with this interpretation, several genes associated with ER signaling and luminal differentiation, including SCUBE2, NAT1, RERG, and PIP, were downregulated in Group 1 tumors. These findings suggest that the tumor-positive/stroma-negative CD138 phenotype may be associated with a less differentiated tumor state and a partial loss of luminal differentiation programs.

One possible explanation linking CD138 expression to the observed transcriptomic pattern is growth factor signaling mediated by CD138 expression. CD138 can retain growth factors, such as FGF, HGF, and VEGF, on the cell surface through its heparan sulfate chains, thereby enhancing receptor-mediated signaling. These signals may activate MAPK and PI3K–Akt–mTOR pathways, which regulate translation initiation and ribosome biogenesis. Persistent CD138 expression in tumor cells may contribute to the transcriptomic changes observed in snoRNA expression and ribosome-related pathways.

The CD138-associated transcriptional program included the upregulation of protein-coding genes, such as S100A7 and CD24, which was strongly correlated with the CD138 signature. Both genes have been implicated in tumor invasion, immune evasion, and metastasis [50,51,52,53,54]. These genes have also been associated with brain metastasis and aggressive tumor behavior [11,55,56], consistent with the transcriptomic pattern observed in Group 1 tumors.

Consistent with these molecular findings, the tumor-positive/stroma-negative CD138 phenotype remained independently associated with RFS after adjusting for established clinicopathological factors, including lymph node status and Ki-67 index. This phenotype was associated with HER2 positivity, nodal metastasis, high HG, and brain metastases, although the number of brain metastases was small. Furthermore, we clarified that the observation of brain metastasis exclusively in Group 1 should be interpreted with caution, given the limited number of cases; therefore, these findings must be validated in larger cohorts.

Syndecan-1 has been implicated in tumor invasion and metastatic adaptation through the modulation of growth factor signaling and ECM interactions [56,57].

Furthermore, we expanded our discussion to consider the potential involvement of CD138-mediated signaling pathways, including the PI3K–Akt–mTOR axis. However, this proposed mechanism remains speculative, and further in vitro functional studies are required to validate the causal link and clarify its role in translational reprogramming. Future studies using spatial transcriptomics may provide a more precise understanding of the relationship between compartment-specific CD138 expression and associated transcriptomic alterations, enabling direct spatial linkage between tumor cells, stromal components, and molecular signatures.

In addition, the potential involvement of the immune microenvironment, including tumor-infiltrating lymphocytes, should be considered. The interplay between CD138-defined tumor states and immune cell infiltration may contribute to the observed differences in tumor behavior, warranting further investigation.

One limitation of this study is the relatively modest cohort size used for multi-group biomarker analysis. While the present findings highlight the potential clinical significance of compartment-specific CD138 expression, further validation in larger, independent cohorts is warranted to confirm the robustness, reproducibility, and generalizability of these results.

Taken together, our findings suggest that compartment-specific CD138 expression is associated with a tumor state characterized by alterations in RNA-related pathways and a partial loss of luminal differentiation programs. These molecular alterations may contribute to tumor aggressiveness; however, their relationship with metastasis and treatment response requires further study.

## 5. Conclusions

In this study, we show that the tumor-positive/stroma-negative CD138 expression pattern is associated with poorer RFS and transcriptomic features related to snoRNA expression and RNA processing in invasive breast cancer.

These findings suggest that compartment-specific CD138 evaluation may have prognostic value, warranting further validation in larger independent cohorts and mechanistic studies.

## Figures and Tables

**Table 1:** The clinicopathological characteristics of the study cohort.

Factor		n (%)
Age (year)	Median	59
	Range	27–82
	<40	10 (9.0)
	40≤	101 (91.0)
T	T1	60 (54.1)
	T2	45 (40.5)
	T3	5 (4.5)
	T4	1 (0.9)
N	N0	82 (73.9)
	N1	18 (16.2)
	N2	6 (5.4)
	N3	5 (4.5)
HG	HG1	34 (30.6)
	HG2	36 (32.4)
	HG3	41 (36.9)
Ki67	<20	42 (37.8)
	20≤	69 (62.2)
Subtype	HR+HER2−	64 (57.7)
	HR+HER2+	8 (7.2)
	HR−HER2+	16 (14.4)
	HR−HER2−	23 (20.7)
RFS	No metastasis	89 (80.2)
	Metastasis	22 (19.8)
Adjuvant therapy	Radiotherapy	69 (62.2)
	Chemotherapy	55 (49.5)
	Endocrine therapy	62 (55.9)
	Anti-HER2 therapy	16 (14.4)

The baseline patient and tumor characteristics of the 111 primary breast cancer cases included in this study. The variables included age, tumor size (T stage), lymph node status (N stage), histological grade (HG), Ki-67 index, hormone receptor (HR) status, HER2 status, recurrence events, and postoperative therapies. Abbreviations: HG, histological grade; HR, hormone receptor; HER2, human epidermal growth factor receptor 2; RFS, recurrence-free survival.

**Table 2:** CD138 immunohistochemical staining patterns.

Area	Factor	n (%)
Tumor	Negative	42 (37.8)
	Weak	17 (15.3)
	Moderate	12 (10.8)
	Strong	20 (18)
Tumor membranous	−	76 (68.5)
	+	35 (31.5)
Tumor cytoplasmic	−	43 (38.7)
	+	68 (61.3)
Tumor nuclear	−	107 (96.4)
	+	4 (3.6)
Stroma	Negative	84 (75.7)
	Weak	5 (4.5)
	Moderate	3 (2.7)
	Strong	6 (5.4)

CD138 expression patterns and staining intensity were based on previously reported criteria [21,22], in reference to a semi-quantitative scoring system integrating staining intensity and the proportion of positive cells described by Lennartz et al. [24], with minor modifications. The number of samples corresponding to each staining category is shown.

**Table 3:** The classification of CD138 immunohistochemical expression patterns.

Classification Group	Group 0	Group 1	Group 2	Group 3
Tumor	−	+	−	+
Stroma	−	−	+	+
Numbers	45	35	19	12

Group 0: tumor-negative/stroma-negative; Group 1: tumor-positive/stroma-negative; Group 2: tumor-negative/stroma-positive; Group 3: tumor-positive/stroma-positive.

**Table 4:** The association between CD138 classification groups and CD138 transcript expression levels (TPM).

TPM	Group 0	Group 1	Group 2	Group 3
Low	6	15	8	19
High	6	20	11	26

The cutoff value for TPM expression was determined using receiver operating characteristic (ROC) curve analysis with recurrence-free survival (RFS) as the endpoint. No significant association was observed between the TPM expression and CD138 classification groups (chi-square test, χ^2^ = 0.254, df = 3, *p* = 0.968). Abbreviations: TPM, transcripts per million.

**Table 5:** Differentially expressed RNAs identified based on CD138 immunohistochemical expression patterns. (**a**) RNAs were significantly upregulated in Group 1 (tumor-positive/stroma-negative). (**b**) RNAs were significantly downregulated in Group 1 (tumor-positive/stroma-negative group).

(**a**)			
**Rownames (Data)**	**Gene Name**	**Gene Type**	***p*-Value**
ENSG00000199293	SNORA21	snoRNA	0.0415115
ENSG00000206834	SNORA1	snoRNA	0.043876146
ENSG00000207112	SNORA25	snoRNA	0.045203849
ENSG00000207118	SNORD14D	snoRNA	0.012961092
ENSG00000207165	SNORA70	snoRNA	0.009010414
ENSG00000207181	SNORA14B	snoRNA	0.007170734
ENSG00000207344	SNORA22C	snoRNA	0.048681431
ENSG00000221066	SNORD111	snoRNA	0.045965701
ENSG00000221514	SNORD111B	snoRNA	0.012779495
ENSG00000252699	SNORA21B	snoRNA	0.001568441
ENSG00000277985	SNORA67	snoRNA	0.040745568
ENSG00000252906	SCARNA3	scaRNA	0.01370412
ENSG00000200537	RNY4P6	misc_RNA	0.034323474
ENSG00000252355	RN7SKP287	misc_RNA	0.000379685
ENSG00000258232	0	lncRNA	0.011300409
ENSG00000272114	0	lncRNA	0.005735046
ENSG00000207029	RNU6-43P	snRNA	0.024349242
ENSG00000223336	RNU2-6P	snRNA	0.039879665
ENSG00000143556	S100A7	protein_coding	0.035694481
ENSG00000156689	GLYATL2	protein_coding	3.27328 × 10^−5^
ENSG00000272398	CD24	protein_coding	1.88457 × 10^−5^
(**b**)			
**Rownames (Data)**	**Gene Name**	**Gene Type**	***p*** **-Value**
ENSG00000212195	U3	snoRNA	0.007025747
ENSG00000271817	U3	snoRNA	0.000177356
ENSG00000278249	SCARNA2	scaRNA	0.00041631
ENSG00000200065	Y_RNA	misc_RNA	0.004822455
ENSG00000200090	Y_RNA	misc_RNA	0.001984721
ENSG00000200502	Y_RNA	misc_RNA	0.029705185
ENSG00000201496	RN7SKP275	misc_RNA	0.001974826
ENSG00000202417	Y_RNA	misc_RNA	0.018438651
ENSG00000202522	Y_RNA	misc_RNA	0.047206429
ENSG00000203286	Metazoa_SRP	misc_RNA	0.032145978
ENSG00000238324	RN7SKP198	misc_RNA	0.005390875
ENSG00000239942	RN7SL394P	misc_RNA	0.000624811
ENSG00000244218	RN7SL81P	misc_RNA	0.001850411
ENSG00000252621	Y_RNA	misc_RNA	0.038943206
ENSG00000272197	RN7SL803P	misc_RNA	0.000998156
ENSG00000273974	0	misc_RNA	0.031202327
ENSG00000274963	RN7SL600P	misc_RNA	0.00780226
ENSG00000276089	0	misc_RNA	0.026045989
ENSG00000276096	Metazoa_SRP	misc_RNA	0.009464143
ENSG00000175746	LINC02915	lncRNA	0.029813027
ENSG00000197308	GATA3-AS1	lncRNA	0.005752834
ENSG00000203497	PDCD4-AS1	lncRNA	0.000147504
ENSG00000213904	LIPE-AS1	lncRNA	0.003981315
ENSG00000224559	LINC01087	lncRNA	0.018170724
ENSG00000227392	HPN-AS1	lncRNA	0.000102862
ENSG00000228113	0	lncRNA	0.002414605
ENSG00000228606	DCAF8-DT	lncRNA	0.001413829
ENSG00000230184	SMYD3-IT1	lncRNA	0.005751595
ENSG00000232085	0	lncRNA	0.030106957
ENSG00000232536	0	lncRNA	0.001484405
ENSG00000234160	0	lncRNA	0.025132205
ENSG00000244265	SIAH2-AS1	lncRNA	0.001578035
ENSG00000244491	0	lncRNA	0.002462311
ENSG00000248335	0	lncRNA	0.01796676
ENSG00000248779	0	lncRNA	0.003054656
ENSG00000249306	LINC01411	lncRNA	0.000321652
ENSG00000251141	MRPS30-DT	lncRNA	1.84835 × 10^−5^
ENSG00000254676	0	lncRNA	0.002004225
ENSG00000254815	LMNTD2-AS1	lncRNA	0.001293679
ENSG00000256364	0	lncRNA	0.045775399
ENSG00000257386	0	lncRNA	0.029069361
ENSG00000259175	0	lncRNA	1.03738 × 10^−5^
ENSG00000259269	0	lncRNA	0.045791785
ENSG00000259345	0	lncRNA	0.012564516
ENSG00000259447	0	lncRNA	0.008978821
ENSG00000259642	ST20-AS1	lncRNA	6.45763 × 10^−5^
ENSG00000260372	AQP4-AS1	lncRNA	0.041262915
ENSG00000261505	0	lncRNA	0.000536122
ENSG00000262119	0	lncRNA	0.002303181
ENSG00000263618	0	lncRNA	0.002146189
ENSG00000263677	0	lncRNA	2.12098 × 10^−5^
ENSG00000263924	0	lncRNA	0.043806185
ENSG00000265369	PCAT18	lncRNA	8.53622 × 10^−8^
ENSG00000267395	DM1-AS	lncRNA	0.000483251
ENSG00000272335	0	lncRNA	1.31311 × 10^−8^
ENSG00000272661	0	lncRNA	0.009683057
ENSG00000274259	SYNGAP1-AS1	lncRNA	0.002058406
ENSG00000275894	0	lncRNA	0.00512729
ENSG00000003989	SLC7A2	protein_coding	0.001272185
ENSG00000120251	GRIA2	protein_coding	0.025925214
ENSG00000134533	RERG	protein_coding	0.000121022
ENSG00000141424	SLC39A6	protein_coding	2.22126 × 10^−6^
ENSG00000150667	FSIP1	protein_coding	0.0406572
ENSG00000153002	CPB1	protein_coding	6.03791 × 10^−8^
ENSG00000154099	DNAAF1	protein_coding	0.006580799
ENSG00000159763	PIP	protein_coding	0.003116937
ENSG00000162078	ZG16B	protein_coding	0.004422205
ENSG00000164128	NPY1R	protein_coding	0.04102273
ENSG00000168993	CPLX1	protein_coding	0.000792682
ENSG00000171428	NAT1	protein_coding	0.000448024
ENSG00000173917	HOXB2	protein_coding	0.004842339
ENSG00000175356	SCUBE2	protein_coding	0.00768095
ENSG00000188001	TPRG1	protein_coding	5.08394 × 10^−6^
ENSG00000196090	PTPRT	protein_coding	0.012968594
ENSG00000196136	SERPINA3	protein_coding	0.003046807
ENSG00000198077	CYP2A7	protein_coding	1.90032 × 10^−5^
ENSG00000198650	TAT	protein_coding	1.5222 × 10^−9^
ENSG00000203697	CAPN8	protein_coding	0.003348308
ENSG00000206013	IFITM5	protein_coding	0.000464115
ENSG00000215910	C1orf167	protein_coding	0.049644545

The expression values are shown as log_2_(TPM + 1). Abbreviations: TPM, transcripts per million.

**Table 6:** The annotation of snoRNAs identified in Group 1 (tumor-positive/stroma-negative).

Gene Symbol	Host Gene	Box Type (H/ACA, C/D)	Target rRNA and Nucleotide Position	Modification Type (Ψ or 2′-O-Methylation)	Family Status
SNORA21/21B	RPL23	Box H/ACA	28S: 4401, 4470	Ψ	family
SNORA1	TAF1D	Box H/ACA	28S: 4441	Ψ	single
SNORA25	TAF1D	Box H/ACA	28S: 801, 814	Ψ	single
SNORD14D	HSPA8	Box C/D	18S: 462	2′-O-Me	single
SNORA70	RPL10	Box H/ACA	18S: 1232, 1692	Ψ	single
SNORA14B	TOMM20	Box H/ACA	18S: 681, 966	Ψ	single
SNORA22C	CCT6P3	Box H/ACA	18S: 918	Ψ	single
SNORD111/111B	SF3B3	Box C/D	28S: 3823	2′-O-Me	family
SNORA67	EIF4A1	Box H/ACA	18S: 1445	Ψ	single

The table summarizes snoRNAs that were significantly upregulated in Group 1 based on the CD138 immunohistochemical classification. For each snoRNA, the corresponding host gene, box class (H/ACA or C/D), target rRNA and nucleotide position, modification type (pseudouridylation [Ψ] or 2′-O-methylation), and family status are shown. Family status indicates whether a snoRNA belongs to a paralogous gene family or exists as a single-copy gene family. rRNA targets and nucleotide positions are based on canonical human rRNA annotation (18S or 28S).

**Table 7:** A comparison of clinicopathological characteristics between the CD138 tumor-positive/stroma-negative group (Group 1) and all other groups.

Variable	Group 1	Others	*p*-Value
Age (<40)	6	4	0.0749
T (T23)	16	35	0.951
N (N123)	17	15	0.00064
HG (HG3)	17	24	0.036
Ki67 (10%≤)	21	48	0.804
HR+	18	54	0.0437
HER2+	14	10	0.00218
Brain meta	4	0	0.0098 *
Radiotherapy	21	48	0.804
Chemotherapy	19	36	0.444
Endocrine therapy	15	47	0.077
Anti-HER2 therapy	10	6	0.00116
TPM (CD138: high)	20	43	0.974

Group differences were evaluated using the chi-square or the Fisher exact test, as appropriate. * indicates that Fisher’s exact test was used. Underlined values indicate *p* < 0.05.

**Table 8:** The association between the CD138 expression group (Group 1), clinicopathological factors, and prognostic analysis.

(**a**)								
**Factor**	**Univariate**				**Multivariate**			
	**HR**	**95% CI**		***p*-Value**	**HR**	**95% CI**		***p*-Value**
		**Lower Limit**	**Upper Limit**			**Lower Limit**	**Upper Limit**	
Age	0.2732	0.0992	0.7522	0.01204	0.2014	0.05244	0.7735	0.0196
T	3.882	1.518	9.927	0.004639	1.942	0.6052	6.23	0.2645
N	4.581	1.954	10.74	0.0004618	4.763	1.717	13.21	0.002711
HG	2.123	0.9165	4.918	0.07899	0.9324	0.2381	3.65	0.9199
Ki67	3.025	1.02	8.969	0.04588	3.789	1.052	13.65	0.04164
HR	0.493	0.2134	1.139	0.09794	0.8687	0.1665	4.532	0.8674
HER2	1.143	0.421	3.102	0.7933	1.063	0.2429	4.654	0.9351
CD138	4.877	1.994	11.93	0.0005157	6.13	2.081	18.06	0.001005
Radiotherapy	0.6827	0.2948	1.581	0.3729	0.9715	0.3462	2.726	0.9563
Chemotherapy	1.124	0.4844	2.607	0.7858	0.4687	0.1586	1.385	0.1705
Endocrine therapy	0.3931	0.1648	0.9374	0.03523	0.9497	0.1915	4.709	0.9496
Anti-HER2 therapy	0.5659	0.132	2.427	0.4434	0.1325	0.01492	1.176	0.0696
(**b**)								
**Factor**			**rho**			***p*** **-Value**		
Age			−0.012			0.901		
N			0.322			0.000578		
Ki67			−0.113			0.24		

(**a**) Univariate and multivariate Cox proportional hazards regression analyses were performed to evaluate the prognostic factors for recurrence-free survival (RFS). Hazard ratios (HRs), 95% confidence intervals (CIs), and *p*-values were estimated using the Cox proportional hazards regression. The analysis included 111 patients, among whom 22 experienced recurrence events during the follow-up period. The variables were defined as follows: age, <40 years; T stage, T2–T3; N stage, N1–N3; HG, histological grade 3; Ki-67, high expression ≥ 20%; HR, hormone receptor-positive; HER2, HER2 positive; CD138, tumor-positive/stroma-negative phenotype. Brain metastases were not included in the multivariate model because all brain metastases occurred in the CD138 tumor-positive/stroma-negative group. Statistical significance was set at *p* < 0.05. Underlined values indicate *p* < 0.05 (**b**) The correlation between Group 1 CD138 expression and clinicopathological factors. Spearman rank correlation coefficient was used to evaluate associations between Group 1 CD138 classification and clinicopathological factors that were significant in multivariable Cox regression analysis (Table 8b), including age, lymph node status (N stage), and Ki-67 index. A weak correlation was observed between CD138 classification and lymph node status, whereas no significant correlations were observed between age or Ki-67 index. Underlined values indicate *p* < 0.05.

## Data Availability

The RNA sequencing data generated in this study are openly available in the DNA Data Bank of Japan (DDBJ) Sequence Read Archive under the BioProject accession numbers PRJDB37924 and PRJDB40174.

## Supplementary Materials

The following supporting information can be downloaded at https://www.mdpi.com/article/10.3390/cancers18101539/s1. Supplementary Table S1. Immunohistochemical Scoring Criteria for CD138 (Syndecan-1).

## Abbreviations

The following abbreviations are used in this manuscript.

ASCO	American Society of Clinical Oncology
BP	Biological Process
CAP	College of American Pathologists
CD138	Cluster of Differentiation 138 (Syndecan-1)
CI	Confidence Interval
DAVID	Database for Annotation, Visualization and Integrated Discovery
DEG	Differentially Expressed Gene
ECM	Extracellular Matrix
EMT	Epithelial–Mesenchymal Transition
ER	Estrogen Receptor
FDR	False Discovery Rate
FFPE	Formalin-Fixed Paraffin-Embedded
FGF	Fibroblast Growth Factor
GO	Gene Ontology
GSVA	Gene Set Variation Analysis
HER2	Human Epidermal Growth Factor Receptor 2
HG	Histological Grade
HGF	Hepatocyte Growth Factor
HR	Hazard Ratio
HR status	Hormone Receptor Status
IDC	Invasive Ductal Carcinoma
IHC	Immunohistochemistry
KEGG	Kyoto Encyclopedia of Genes and Genomes
lncRNA	Long Non-Coding RNA
MAPK	Mitogen-Activated Protein Kinase
ncRNA	Non-Coding RNA
PgR	Progesterone Receptor
PI3K	Phosphoinositide 3-Kinase
ROC	Receiver Operating Characteristic
RFS	Recurrence-Free Survival
RNA	Ribonucleic Acid
RNA-seq	RNA Sequencing
rRNA	Ribosomal RNA
snoRNA	Small Nucleolar RNA
SRP	Signal Recognition Particle
ssGSEA	Single-Sample Gene Set Enrichment Analysis
TPM	Transcripts Per Million
UICC	Union for International Cancer Control
VEGF	Vascular Endothelial Growth Factor
Ψ	Pseudouridylation
