# Supplementary material for: Compartment-Specific CD138 Expression Defines an Aggressive Breast Cancer Phenotype with Distinct Transcriptomic Features
Source: Cancers (Basel). 2026 May 9;18(10):1539. doi: 10.3390/cancers18101539 (PMC13204842; doi:10.3390/cancers18101539)
Supplement: Supplementary file 1 [file cancers-18-01539-s001.zip › cancers-4266808-supplementary.pdf]

cancers

MDPI

Supplementary Material: Compartment-Specific CD138 Expression Defines an Aggressive Breast Cancer Phenotype with Distinct Transcriptomic Features

Kyoko Asai, Takahiro Hasebe, Masahiro Ohara, Masataka Hirasaki, Kazuo Matsuura, Hiroshi Ishiguro, Akihiko Osaki and Toshiaki Saeki

Table S1.

	Tumor cells		Stroma	
	Percentage of stained tumor cells	Staining intensity	Percentage of stained stromal area	Staining intensity
Negative	0%	0	0%	0
Weak	≦70%	1+	≦70%	1+
	≦30％	2+	≦30％	2+
Moderate	＞70％	1+	＞70％	1+
	31-70％	2+	31-70％	2+
	≦30％	3+	≦30％	3+
Strong	＞70％	2+	＞70％	2+
	＞30％	3+	＞30％	3+

 https://doi.org/10.3390/18101539
